# Comparison of knowledge scores of medical students in problem-based learning and traditional curriculum on public health topics

**DOI:** 10.1186/1472-6920-5-7

**Published:** 2005-02-10

**Authors:** Erol Gurpinar, Berna Musal, Gazanfer Aksakoglu, Reyhan Ucku

**Affiliations:** 1 Akdeniz University School of Medicine Department of Medical Education, Antalya Turkey; 2 Dokuz Eylul University School of Medicine Department of Medical Education, Izmir Turkey; 3 Dokuz Eylul University School of Medicine Department of Public Health, Izmir Turkey

## Abstract

**Background:**

The purpose of the study was to compare the knowledge scores of medical students in Problem-based Learning and traditional curriculum on public health topics.

**Methods:**

We planned a cross-sectional study including the fifth and sixth year medical students of Dokuz Eylul University in Turkey. The fifth year students (PBL group, n = 56) were the pioneers educated with PBL curriculum since the 1997–1998 academic year. The sixth year students (traditional education group, n = 78) were the last students educated with traditional education methods. We prepared 25 multiple-choice questions in order to assess knowledge scores of students on selected subjects of Public Health. Our data were collected in year 2002.

**Results:**

Mean test scores achieved in PBL and traditional groups were 65.0 and 60.5 respectively. PBL students were significantly more successful in the knowledge test (p = 0.01). The knowledge scores of two topics were statistically higher among PBL students. These topics were health management and chronic diseases.

**Conclusion:**

We found that mean total evaluation score in the PBL group was 4.5 points higher than in the traditional group in our study. Focusing only on the knowledge scores of students is the main limitation of our study. Upon the graduation of the first PBL students in the 2002–2003 academic year, we are planning additional studies regarding the other functions of a physician such as skill, behaviour and attitude.

## Background

During the last 25 years, ideas concerning the aim, structure and system of medical education have been discussed. Debates generally have arisen from the perception that medical education couldn't serve the purpose of improving health standards of the communities [1].

"Health for All" was adopted in 1977 and launched at the Alma Ata Conference in 1978 to underline the fact that large numbers of people and even whole countries were not enjoying an acceptable standard of health [2]. In order to achieve the goal of "Health for All" and to improve the health standards, medical schools must provide physicians who are familiar with the community and its health problems, their prevention and solutions. Then their curriculum must be expedient to this goal [3,4]. World Health Organization (WHO) also emphasizes the fact that medical students must be educated considering the health needs of the population in which they live [5].

In the Edinburgh Declaration of the World Medical Association in 1988, similar problems were mentioned and the purpose of the medical education was declared as training physicians capable of improving communities' health standards. This declaration suggested that medical education should be focused on common health problems of the large communities, and the medical school curriculum should be restructured according to the health requirements of the community. According to the declaration, medical students must gain professional skills and social values in addition to theoretical knowledge and the principle of lifelong medical education should be adopted [6].

The ideas and suggestions mentioned above have aroused strong winds of change in the medical education arena. Mc Donald et al. from Mc Master University determined an approach based on the community's main health problems and stressed the importance of focusing on these problems while designing their medical school's curriculum [7].

Since then, this approach has been adopted by many medical schools all over the world. The schools which designed their curriculum according to the priority health problems of the community, managed to raise the physicians' awareness of their community and the preventive measures and solutions of their main health problems.

In Turkey, problems of medical education have been discussed since early 1970s. Several studies showed that the goals of medical education did not overlap with the health requirements of the Turkish community. The education of health professionals was abstracted from the realities of the country. In 1990s Turkish Parliament and Turkish Medical Association determined and reported the difficulties of medical education. In a 1991 report of the Turkish Parliament, the facts that the number of qualified physicians who were trained according to the health needs of the country was limited and that this number was not sufficient to improve its health standards were underlined. Several deans from different medical schools of the country contributed to Turkish Parliament's study and reported that a greater importance should be given to the health problems of the population while planning the educational programs and the medical education should not be restricted to the university hospitals [8].

In The Turkish Medical Association's report the fact that medical education was not relevant to health needs of the country was emphasized. New medical graduates were not fully aware of common national health problems. The recommendations of the Turkish Medical Association to improve the health standards of the Turkish population were; training the general practitioners capable of working effectively in the primary health care and restructuring the medical education on a community basis and implementing Problem-based Learning methods [9].

International developments and the reports of Turkish Parliament and Turkish Medical Association led the faculty of Dokuz Eylül University School of Medicine (DEUSM) to seek solutions to the problems mentioned in the reports. As a result, Problem-based Learning (PBL) a more active and student-centred learning- was adopted and launched in the 1997–98 academic year. One of the main features of the education program was its relevancy to the philosophy of community-based medical education [10].

The curriculum of DEUSM was structured considering social, biological, behavioural and ethics objectives of medical education. The curriculum was structured in a modular system and adopted to a spiral configuration providing horizontal and vertical integration. During the first three years of undergraduate education, PBL sessions are the main focus of a modular structure. The weekly schedule of a module allowed for all the educational activities such as PBL sessions, lectures, field studies, communication skills and clinical skills courses lectures existing one hour a day in the weekly program support the PBL sessions and independent learning [11].

PBL sessions were based on written problems, which are likely to happen in real life. Special emphasis was also given to the integration of knowledge, acquisition of professional and moral values and to the development of communication skills.

Medical knowledge and practical skills that a physician is supposed to have were on the basis of the advice of Turkish Medical Association and the faculty departments. The Department of Public Health also contributed to the education program by setting social standards and determining the most important health problems of the community.

PBL Curriculum of DEUSM aimed to teach the students the main health problems of the community, their prevention and ways of treatment.

Public Health topics of Dokuz Eylül University School of Medicine consists of;

• Holistic approach in health,

• Basic principles of Public Health,

• Personal and social points of view on health events,

• Bio-psychosocial (holistic) approach to any individual,

• Principles of preventive medicine,

• Structure and mechanisms of national health organization,

• Demographic structure and trends, factors affecting them,

• Basic principles of planning and conducting a scientific research on health,

• Sound knowledge on leading health problems of the country, personal and social approaches for their solutions,

• Environmental and occupational factors threatening community health and their prevention.

Cases in the scenarios of the PBL modules were selected among common and important health problems, for which early diagnosis or prevention is possible. Lectures and small group studies with students were also organized to contribute to the educational effectiveness of the modules. Public Health topics of the medical education may be achieved more easily when theoretical knowledge and practical skills are complemented by field studies [12]. It is recommended to start such activities as early as possible and to continue them during medical education. In DEUSM Public Health perspective, objectives of each academic year were determined and relevant field study programs were developed to contribute these objectives. These programs were put into practice beginning from the beginning of the medical education.

Prior to the implementation of PBL curriculum in the 1997–1998 academic year, lectures on Public Health were presented to the first, the third and the last year students by the faculty members of the Department of Public Health. Lectures on bio-statistics and research methods were given weekly throughout the first year. The other topics of Public Health were held in 72-hour Public Health Courses at the end of the third year [13]. In the new curriculum public health subjects were held in PBL sessions. Each PBL scenario had at least one chapter associated with public health issues. Another difference between traditional and problem based education methods was changing roles of the students and teaching. Traditional education was teacher based and the students were passive receivers while the lecturer was giving information. But in PBL method, roles were exchanged and the sessions were carried out by noninformative teachers and more active students.

Comparison of old and new curriculum using some measurement tools is mandatory to observe the effects of innovations. In the literature, the determination of students' performances in scientific or licensing examinations was used to compare the efficiency of traditional education and PBL. Nandi P. et al. reviewed the studies and meta-analyses comparing PBL and traditional lecture-based education methods. In meta-analysis of the data published between 1980–1999, they concluded that PBL helped students show slightly but not significantly better performance than the others on clinical examinations [14]. Similar results were reported by Albanese M. et al., in a meta-analytic study evaluating published data between 1972–1992 [15].

Blake et al. compared formerly lecture-based educated and recently Problem-based educated graduates of Missouri-Columbia School of Medicine concerning their performances on medical licensing examinations. They reported that mean scores achieved on these examinations were better among graduates of PBL, but the difference between old and new graduates' scores was not statistically significant [16].

Some other studies have attempted to compare students' performances on special areas of medicine instead of general evaluation. Antepohl and Herzig conducted a randomized controlled study among the students who enrolled for the course of basic pharmacology at the University of Cologne. They randomly divided the students into two groups of PBL and traditional lecture based learning in order to compare their final examination scores. They could not find any significant difference between the two groups. However, in short essay questions there was a tendency towards higher scores among the students in the PBL group. The authors also found that the PBL students reached almost identical scores in their multiple choice questions and their short essay questions whereas the students who had been in the lecture based group scored significantly lower scores in their short essays than in their multiple choice questions [17].

In a multi-centric study conducted by Schmidt et al., comparison of PBL and lecture based learning students showed that PBL students had higher knowledge scores on the areas of primary care services, psychological health, collaboration of different sectors on health and occupational ethics [18].

The purpose of our study was to compare the knowledge scores of medical students in PBL and traditional curriculum on public health topics.

## Methods

We planned a cross-sectional study including the fifth and sixth year medical students of DEUSM. The fifth year students (PBL students) were the pioneers educated with PBL curriculum since the 1997–1998 academic year. The sixth year students (traditional education group) were the last students educated with traditional education methods. The knowledge scores of students on Public Health topics were evaluated. In both of the PBL and Traditional curriculum, all the knowledge acquired in the first five years of the school was reviewed during the two-month Public Health internship period in the sixth year. Since this period may remind the students of some issues which may have been previously forgotten, we decided to exclude the sixth year students who have completed their internship period. 56 fifth year students and 78 sixth year students who have not so far completed their internship period in the Department of Public Health were included in our study. Participation rates were 96.4% (54 out of 56 students) in the fifth year and 100% (all of the students) in the sixth.

Before the application of the inquiry form, the purpose of the study was explained to the students and their oral consents were obtained.

We analyzed the knowledge scores of the two groups of students' on Public Health issues. PBL and traditional programs were the independent variables. Descriptive variables were age and gender.

By reviewing a five yearlong section of educational programs, we determined that nine Public Health main topics were common to both PBL and traditional programs. The main topics were communicable diseases, epidemiology, mother and child health, health management, chronic diseases, occupational health, nutritional principles in community, demography and environmental health.

We prepared 25 multiple-choice questions in order to assess knowledge scores of students on selected subjects. The number of questions related to each topic was proportional to the time allocated for each of the topic in the curriculum. The content validity of the questions was tested by consulting experts in relevant fields. All the data were collected between February and March 2002. Scoring procedure was implemented over "100 points" where each correct answer was scored "four points" and each wrong answer was scored "zero point".

Data were subjected to statistical analysis by the chi-square test and the t-test in SPSS 10.0.

## Results

Overall mean age was 23.6 ± 2.1 (21–45) years. The rates of male and female students were 55.4 % and 44.6 % respectively. There were no statistically significant differences between the two groups regarding mean ages, gender distribution or other personal variables.

Mean scores achieved at the 25 question-test were 65.0 in PBL group and 60.5 in the traditional group. Students in the PBL group were significantly more successful in the knowledge test (Table-1).

**Table 1:** Comparison of mean scores of the students in PBL and traditional programs

**Topics**	**Max. point for each topic**	**PBL**		**Traditional**		**t**	**p**
				
		**Mean Score**	**(±) SD**	**Mean score**	**(±) SD**		
Communicable diseases	20	12.5	4.32	12.3	3.74	0.290	0.77
Epidemiology	20	6.2	4.23	5.4	4.13	0.990	0.32
Mother and child health	20	14.9	3.89	15.5	3.70	-0.851	0.39
Health management	12	9.6	2.74	7.7	3.34	3.447	**0.00**
Chronic diseases	8	6.7	2.17	5.3	2.78	3.255	**0.00**
Occupational health	8	6.1	2.54	5.7	2.46	0.743	0.45
Nutritional principles in community	4	1.9	2.01	2.0	2.01	-0.416	0.67
Demography	4	3.3	1.50	2.9	1.75	1.257	0.21
Environmental health	4	3.6	1.17	3.3	1.45	1.070	0.28
Total	100	65.0	10.99	60.5	9.22	2.395	**0.01**

The knowledge scores of seven topics were higher among students in PBL curriculum. These topics were communicable diseases, epidemiology, health management, chronic diseases, occupational health, demography and environmental health. Traditional curriculum students were found to be more knowledgeable on two topics; mother and child health and nutritional principles in the community. However, the differences between PBL and traditional students' knowledge scores in only two topics, chronic diseases and health management, were statistically significant (Table-1).

## Conclusions

In our study, we found a statistically significant difference between knowledge scores of PBL and Traditional education groups in favour of the PBL group (Table 1).

The students of the PBL group had higher knowledge scores on 7 of the 9 identified topics. But the difference between mean scores of the groups was statistically significant in only two topics, "health management" and "chronic diseases". The reason of significantly higher knowledge scores among the students in PBL group may be that these students have more opportunities such as observations during field studies, work-shops or presentations to study on these two topics than those in the other group. They experienced a two week training period in a "community health center" at the end of the first year and observed the health center services and prepared a structured form concerning the procedures of health centers. They also studied in "community health centers" as small groups including two students in each fortnightly during their third year in the school and completed comprehensive forms about the topics on which they studied. The reason of better knowledge scores of PBL group on "chronic diseases" may result from the special educational efforts improving the effects of relevant modules on this topic. Actually special learning opportunities were provided for all topics and we were expecting to find a difference on remaining 7 topics too. On the other hand, the students in the traditional education group had slightly higher mean scores about the topics of "mother and child health" and "nutritional principles in community" although the differences between the groups' mean scores were not statistically significant. These knowledge deficiencies among PBL students were already revealed and an additional module was implemented in the curriculum to compensate them. Curriculum of DEUSM is being looked over by curriculum committee continuously and the departments try to make interventions for problematic parts.

We found that the mean total evaluation score in the PBL group was 4.5 points higher than in the traditional group in our study. Actually, we expected a much larger difference between the two groups in favour of PBL students for their education was supported by lectures, small group studies and field studies in addition to the PBL sessions. They also had the advantage of studying on Public Health issues in each year of the school by means of homogenous allocation of the modules and blocks in the first five years instead of accumulation in a short period of time as it was in the traditional curriculum. Therefore, the difference between the evaluation scores of the groups did not meet our expectations although it was statistically significant. The reason for this underachievement of Public Health objectives among our PBL students may be related to both students and PBL tutors. The common perception among the students that they have enough knowledge to say something about social and behavioural aspects of PBL modules lead them to focus on biological objectives more and they do not need to study on social issues in depth. Furthermore, a common misunderstanding among faculty members that achieving the Public Health objectives in PBL is just the responsibility of the Department of Public Health may have led the PBL tutors to withdraw from the responsibility of focusing on these subjects sufficiently. Additionally, when they are less informed or less equipped with supporting material about Public Health objectives, they may not have felt very competent while facilitating their groups by asking appropriate questions.

One assumption of curricular comparison studies, included this one, is that students will do better either in one or the other type of curriculum. However, each curriculum demands different skills and deployment of learning strategies from the students. This is important because, it is well known in the educational literature that not all students do well in one particular learning program and that they do better when the program adapts to their preferred way of learning. The studies of learning styles may shed light in why the differences between performance scores are always so close when medical curricula are compared.

As we mentioned before, in DEUSM, the written problem used in PBL sessions are oriented to biological as well as social and behavioural objectives. In order to achieve all these three objectives the tutors must attach the same importance to each subject and ensure that their groups give enough time and effort for each objective. But when the tutors get inadequate information and support from the experts of the related subjects, they generally focus only on biological objectives and their groups can't manage to integrate all objectives. If the tutors are less sensitive to objectives other than biological ones, then their students will be less motivated to learn and, like their educators, will be equally insensitive to Public Health topics. In order to prevent this, faculty members of the department of Public Health who take place in the scenario committees review the PBL problems regarding Public Health objectives. They make every effort to insure that the Public Health objectives are included while writing the problems and that the tutors are sufficiently informed on these objectives before their sessions. Field Work Committee has been trying to increase students' motivation and raise their awareness on Public Health issues to increase the effectiveness of field studies.

Focusing only on the knowledge scores of students is the main limitation of our study. Upon the graduation of the first PBL students in the 2002–2003 academic year, we are planning additional studies regarding the other functions of a physician such as skill, behavior and attitude.

## Competing interests

The authors declare that they have no competing interests.

## Authors' contributions

EG conceived of the study, participated in the design of the study and drafted the manuscript, BM conceived of the study, participated in the design of the study and coordination, performed the statistical analysis, GA participated in the design of the study and performed the statistical analysis, RU conceived of the study, participated in the design of the study. All authors read and approved the final manuscript.

## Appendix

### Sample questions

#### Chronic diseases

While working in a health center as a general practitioner, you have noticed that hypertension prevalence is high among the people living in the region under your responsibility. Which of the following would be your choice as primary prevention method?

a) I would educate the hypertensive patients on their disease.

b) I would treat the hypertensive patients with antihypertensive drugs.

c) I would send the hypertensive patients to a secondary care hospital for further investigation and treatment.

d) I would educate healthy individuals on risk factors associated with hypertension and prevention methods.

#### Nutritional rules in community

Which of the followings is the most common childhood nutritional disorder in Turkey?

a) Protein calorie deficiency

b) Marasmus

c) Iron deficiency anaemia

d) Rickets

#### Demography

Which of the following is wrong?

a) Demography is a science that analyse the body, structure and differentiations of human populations.

b) The goal of family planning is to decrease current number of population.

c) Dependent population ratio is found by dividing the total number of population younger than fifteen years and older than 65 years of age by the total number of population between 15–65 years of age.

d) Principal of pronatalist population policy is to increase the total number of population.

#### Health Management

Which of the followings is not one of the basic records kept in a health center?

a) Household determination card.

b) Follow-up card for the females between 15–49 years old.

c) Follow-up card for aged individuals.

d) Antenatal and postnatal follow up card.

e) Infant and child follow-up card.

#### Occupational health

Which of the followings is not among the responsibilities of an occupational health unit?

a) Health prevention services in work settings

b) Work safety preventions

c) Following up the health and safety conditions in work settings

d) Preventing any interruption in production

e) Giving outpatient clinic services in work setting.

#### Communicable diseases

An 11 year old girl was bitten by a neighbour-dog while she was playing in her house-garden. Which of the followings is not required as an immediate intervention?

a) To investigate if the dog is vaccinated.

b) To vaccinate the girl for rabies prevention.

c) To clean the wound by soap and water.

d) To apply one dose of tetanus vaccine.

e) To try to understand how the dog bit the girl.

#### Mother and child health

Which of the followings is the most common used effective family planning (contraception) methods?

a) Intrauterine device

b) Withdrawal (coitus interruptus)

c) Combined oral contraceptives

d) Condom

e) Subcutaneous implants

#### Environmental health

Which of the followings best represents the environmental health related responsibilities of a general practitioner who works in a health centre?

a) Waste control and giving education to correct misapplications

b) Analyzing and chlorinating drinking water, control of potable water

c) Controlling and improving the condition of toilets,

d) Coordination of conduction of above mentioned services by auxiliary personnel of health centre, although these services are among the tasks of municipality.

e) All of the statements above are true.

#### Epidemiology

After looking over one-year medical records of an internal medicine outpatient clinic, it was found that 25 % of the diagnoses were Diabetes mellitus. Regarding this result a screening procedure was conducted in the field and Diabetes mellitus prevalance was found 5 %.

Which of the followings can not be the conclusion of above mentioned situation?

I) Outpatient clinic may admit people coming from other regions.

II) Outpatient clinic records represent the health status of the community.

III) One-fourth of the patients have Diabetes mellitus diagnosis.

IV) Field studies are needed to determine the real prevalance of a disease.

a) I, II

b) I, III

c) II, III

d) I, II, IV

e) II, III, IV

## Pre-publication history

The pre-publication history for this paper can be accessed here:


